# Relationship between thymidine kinase 1 before radiotherapy and prognosis in breast cancer patients with diabetes

**DOI:** 10.1042/BSR20192813

**Published:** 2020-04-15

**Authors:** Zhiwu Wang, Wei Zhang, Bingjie Huo, Liang Dong, Jing Zhang

**Affiliations:** 1 Department of Chemoradiotherapy, Tangshan People’s Hospital, Tangshan, China; 2 Department of Radiotherapy, The Affiliated Yantai Yuhuangding Hospital of Qingdao University, Yantai, Shandong, China; 3 Department of Traditional Chinese Medicine, The Fourth Hospital of Hebei Medical University, Shijiazhuang, China; 4 Department of Endocrinology, Tangshan People’s Hospital, Tangshan, Hebei Province, China

**Keywords:** breast cancer, biomarker, prognosis, overall survival

## Abstract

In a retrospective study design, we explored the relationship between serum thymidine kinase 1 (TK1) concentration before radiotherapy and clinical parameters and evaluated the prognostic value of serum TK1 concentration before radiotherapy in breast cancer patients with type 2 diabetes mellitus. The present study finally consisted of 428 breast cancer patients with a mean age of 53.0 years. Compared with low TK1 group, the high TK1 group tended to have larger tumor size (*P*=0.011) and had more lymph node number (*P*=0.021). Significant differences were also observed in clinical stages I, II and III (*P*=0.000). There was no significant difference between TK1 and other clinical parameters. For disease-free survival (DFS), the univariate analysis indicated that the high TK1 increased the risk of poor prognosis (HR = 2.38, 95% CI: 1.64–4.23, *P*=0.000). The Kaplan–Meier curve indicated the high TK1 group was poorer than that in the low TK1 group (*P*=0.002). For the overall survival (OS), similar results were found that the high TK1 was related to poor OS (HR = 1.89, 95% CI: 1.34–3.67, *P*=0.000). The multivariate Cox regression indicated that the TK1 was still associated with DFS (HR = 1.83, 95% CI: 1.22–3.17, *P*=0.001) and OS (HR = 1.63, 95% CI: 1.19–2.08, *P*=0.006). The high pretreatment serum TK1 levels in breast cancer patients were associated with poor OS and DFS. TK1 could be a potential predictive factor in differential diagnosis of poor prognosis from all patients.

## Introduction

The breast carcinoma is one of the world’s most common cancer in women [1]. The incidence of breast cancer has been increased to different extents both in developing and developed countries [2]. The United States and northern Europe are the areas with highest incidence, eastern and southern Europe and South America rank second, while Asia has the lowest incidence, but in recent years, the gap of incidence of breast cancer is gradually shrinking [3,4]. The incidence of breast cancer is also on the rise and tended to be younger. Although the early breast cancer screening and treatment developed level unceasing progress, but the mortality is still at a very high level. It has the second highest mortality rate after lung cancer [5]. Therefore, the questions related to the prognosis of breast cancer has become the clinical and pathology physician concern.

Radiotherapy is one of the important methods for the treatment of breast cancer, but for ‘tumor-free’ patients after operation, there is no effective efficacy detection index during or after radiotherapy, which brings difficulties for the clinical development of individualized treatment plan [6,7]. Hematologic tumor markers have the advantages of high efficiency, convenience and non-invasiveness in the evaluation of therapeutic effect and prognosis monitoring of breast cancer. Thymidine kinase 1 (TK1), a special kinase, catalyzed thymidine to form 1-phosphothymidine acid, which is an essential precursor for deoxyribonucleic acid (DNA) synthesis of cancerous cells [8]. As an internationally recognized marker of abnormal cell proliferation, TK1 can reflect the dynamic cell proliferation *in vivo* [9]. Studies have shown that serum TK1 can be used as a monitoring indicator for adjuvant therapy of breast cancer, but whether it can be applied to the clinical efficacy and long-term prognosis assessment of patients with advanced breast cancer with her-2 positive remains to be further studied [10]. The TK1 is closely related to the process of angiogenesis, tumor proliferation, differentiation, invasion and metastasis of many tumors [11]. The present study will explore the relationship between serum TK1 concentration before radiotherapy and clinical stage, pathological type and molecular typing of breast cancer patients with type 2 diabetes mellitus (T2DM), and evaluate the prognostic value of serum TK1 concentration before radiotherapy in breast cancer patients with T2DM.

## Materials and methods

We retrospectively collected clinical data and follow-up information of breast cancer patients with T2DM from the Tangshan People’s Hospital, The Affiliated Yantai Yuhuangding Hospital of Qingdao University and The Fourth Hospital of Hebei Medical University between October 2012 and March 2018. Criteria for inclusion: (1) the diagnosis of breast cancer was confirmed by pathology gold standard; (2) patients with T2DM diagnosed before or at the same time of diagnosis of breast cancer and the T2DM diagnostic referred to the diagnostic criteria for diabetes; (3) patients were in a good general condition and KPS > 80; (4) patients did not receive any anti-tumor treatment before being diagnosed; (5) the patient’s cardiopulmonary function was normal, blood routine and liver and kidney function were normal; no other tumors, severe inflammatory diseases, cardiovascular diseases; (6) all patients belonged to the stage I/II/III. Criteria for exclusion: patients with KPS < 80; type I diabetes mellitus, incomplete data, stage IV and male patients were excluded. Those who ever received treatments were also excluded. The present study was approved by the Ethics Committee of Tangshan People’s Hospital. The research was carried out in accordance with the World Medical Association Declaration of Helsinki and all subjects provided written informed consent.

### Information and definition

All demographic information and pathology were obtained from medical records. The following data were collected: age (years), body mass index (BMI; kg/m^2^), smoking (defined: defined as current smoking or smoked daily previously [12]; yes vs no), tumor size (≤2 vs >2), lymph node metastases number (accoding to median of number: 0, 1–5, 6–10, >10). According to the pathological staging standard of breast cancer developed by the American Joint Committee on Cancer (AJCC), patients enrolled in the group were divided into pathological type and clinical staging (I, II, III), molecular subtyping: peritumoral vascular invasion (PVI), estrogen receptor (ER), progesterone receptor (PR), Ki-67, type of surgery (radical vs conservative) and chemotherapy regimens (FEC, TEC or AC-T) and endocrine treatment (Yes or No). TK1 detection acquisition subjects in the morning fasting venous blood 3 ml before receiving treatment and after admission to hospital, room temperature after solidification, 2000 r/min, the centrifugal 5 min (r = 20 cm), serum and 20°C save backup, using enzyme-linked immunosorbent method (linked immunosorbent assay enzyme-1, ELISA) to detect serum TK1 levels.

T2DM was diagnosed according to: (1) American Diabetes Association (ADA) that has guidelines for Diabetes. (2) Blood ≥11.0l mmol/l at 2 h after oral glucose tolerance test. (3) Fasting blood glucose (FPG) ≥ 7.0 mmol/l. Fasting: no calorie intake for at least 8 h. (4) In patients with typical hyperglycemia or hyperglycemia crisis symptoms, random blood glucose ≥11.1l mmol/l. In the absence of definite hyperglycemia, the standard should be confirmed by repeated testing [13].

### Radiotherapy

Radiotherapy was performed by linear accelerator (6 MV X-ray and 9 MeV electron ray). The radiotherapy region was determined according to the tumor stage and tumor site of patients. The radiation dose was 50 Gy/25 times/5 weeks after radical resection. After breast preservation, the whole breast was 50 Gy/25 times, and the electron beam supplementation in tumor bed was 10 Gy/5 times.

### Follow-up

Follow-up data were collected by telephone follow-up and patients were sent to the outpatient department or inpatient department each hospital. All patients were followed up at the beginning of radiotherapy for the first case of breast cancer enrolled in the group, and the death date was the end point. The follow-up period was until October 2018. The primary follow-up outcomes were death or recurrence or distant metastasis. Overall survival (OS) was defined as the time from surgery to death and disease-free survival (DFS) was defined as the time from surgery to local recurrence or distant metastasis [14].

### Statistical analysis

All breast cancer patients were divided into two groups according to the median of TK1 (median = 2.45). Quantitative data were expressed using mean ± standard deviation (SD) or median (minimum, maximum) according to the normality of data distribution. The independent-sample *t* test or non-parameter test was used between high TK1 group (>median) and low TK1 group (≤median). The normality test was performed by Kolmogorov–Smirnov method. The category data were expressed using the count and percent. The Chi-square test was used to compare the differences between two groups. The censoring time was defined as the last follow-up time point. The Kaplan–Meier survival curves with log-rank tests and Cox proportional hazard regression analysis were used to compare the OS rate and DFS rate, respectively. The meaningful variables in univariable analysis entered into the multivariate Cox regression to explore the relationship between TK1 and prognosis in breast cancer patients with T2DM. All analyses were performed using the SPSS 20.0 and GraphPad Prism 8.0. *P*<0.05 was considered statistically significant.

## Results

### General characteristics of study population

The present study finally consisted of 428 breast cancer patients with T2DM according to the criteria for inclusion and exclusion. The mean age was 53.0 ± 9.7 years (range: 40–69 years). The smoking rate was 7.7% (33/428). The ratio of lymph node metastases was 50.5% (lymph node number: 0 for 49.5%, 1–5 for 28.0%, 6–10 for 12.4%, >10 for 7.9%). For histological type, 88.7% of them belong to infiltrated type. According to the stage principle, the ratios of stages I, II and III were 27.1, 43.2 and 29.7%, respectively. The absent ratio of PVI was 88.3%. The positive ratios of ER, PR and her-2 were 61.0, 49.5 and 21.7%, respectively. There were 283 patients with >2 cm tumor size (66.1%). There were 233 patients with Ki-67 > 20 (54.4%). Three hundred and ten patients received the radical surgery treatment and 118 patients received the conservative treatment. One hundred and twelve patients selected the 5-fu/Farumorubishin/cyclophosphamide plan and 262 patients chose the Docetaxel + epirubicin + cyclophosphamide plan. Three hundred and twenty seven patients received endocrine therapy. We divided the patients into high TK1 group and low TK1 group with median TK1 (median TK1 = 103.49 ng/ml).

### Relationship between TK1 and clinical parameters

Table 1 presents the relationship between TK1 and clinicopathological characteristics in patients with breast cancer. There were 214 patients for >median group and ≤median group, respectively. There were no significant differences in age (*P*=0.531), BMI (*P*=0.228) and smoking ratio (*P*=0.587) between high TK1 group and low TK1 group. Compared with low TK1 group, the high TK1 group tended to have larger tumor size (*P*=0.011), have more lymph node number distribution (*P*=0.021). Significant differences were also observed in clinical stages I, II and III (*P*=0.000) between two groups and the high TK1 group tended to have advanced clinical stage. There were no significant differences in PVI (*P*=0.071), ER (*P*=0.766), PR (*P*=0.562) and Her-2 positive rate (*P*=0.412) between the two groups. The expression of Ki-67 showed no significant difference (*P*=0.829). There seemed to be no significant differences in treatment methods, including type of surgery (*P*=0.829), chemotherapy regiments (*P*=0.349) and endocrine therapy (*P*=0.425).

**Table 1:** Relationship between TK1 and clinicopathological characteristics in patients with breast cancer

Parameters	>Median (*n*=214)	≤Median (*n*=214)	χ^2^/t	*P*
Age (years)	53.3 ± 10.3	52.7 ± 9.5	0.626	0.531
BMI (kg/m^2^)	22.4 ± 6.2	23.1 ± 5.8	1.206	0.228
Smoking (*n*, %)	18 (8.4%)	15 (7.0%)	0.296	0.587
Tumor size, cm			6.518	0.011
≤2	85 (39.7%)	60 (28.0%)		
>2	129 (60.3%)	154 (72.0%)		
Lymph node number (*n*, %)			11.588	0.021
0	90 (42.1%)	122 (57.0%)		
1–5	69 (32.2%)	60 (28.0%)		
6–10	33 (15.4%)	20 (9.4%)		
>10	22 (10.3%)	12 (5.6%)		
Pathology stage			33.261	0.000
I	32 (15.0%)	84 (39.3%)		
II	103 (48.1%)	82 (38.3%)		
III	79 (36.9%)	48 (22.4%)		
Histological type			2.373	0.305
Infiltrated type	195 (91.1%)	185 (86.4%)		
Non-infiltrated type	12 (5.6%)	19 (8.9%)		
Others	7 (3.3%)	10 (1.7%)		
PVI (absent, %)	183 (85.5%)	195 (91.1%)	3.261	0.071
ER positive (*n*, %)	129 (60.3%)	132 (61.7%)	0.088	0.766
PR positive (*n*, %)	109 (50.9%)	103 (48.1%)	0.336	0.562
Her-2 positive (*n*, %)	43 (20.1%)	50 (23.4%)	0.673	0.412
Ki-67 > 20	117 (54.7%)	116 (54.2%)	0.009	0.923
Type of surgery			0.047	0.829
Radical	154 (72.0%)	156 (75.1%)		
Conservative	60 (28.0%)	58 (24.9%)		
Chemotherapy (*n*, %)	196 (91.6%)	199 (88.9%)	0.295	0.587
Chemotherapy regimens			2.108	0.349
None	28 (13.1%)	26 (12.1%)		
FEC	62 (29.0%)	50 (23.4%)		
TEC or AC-T	124 (57.9%)	138 (64.5%)		
Endocrine therapy			0.635	0.425
Yes	160 (74.8%)	167 (78.0%)		
No	54 (25.2%)	47 (22.0%)		

### Relationship between TK1 and prognosis

**Figure 1:**
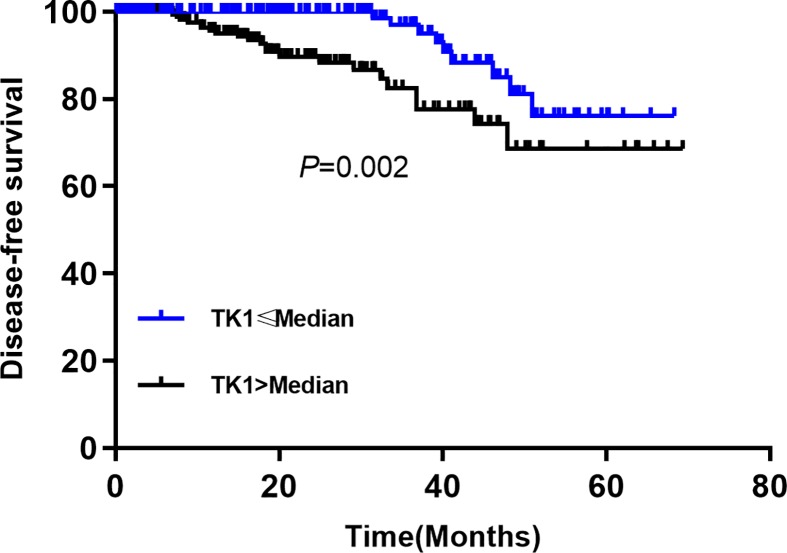
Comparison of DFS between high TK1 and low TK1 group in breast cancer patients with T2DM

**Figure 2:**
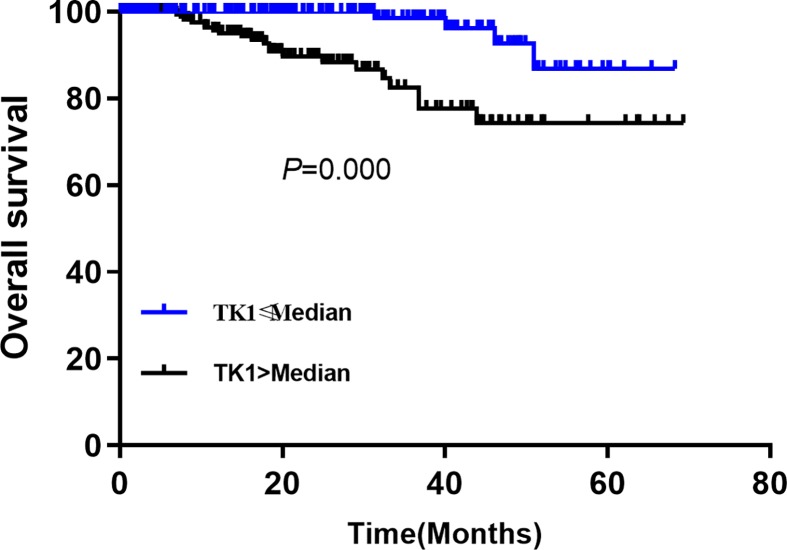
Comparison of OS between high TK1 and low TK1 groups in breast cancer patients with T2DM

The univariate (Table 2) and multivariate Cox regression (Table 3) analyses were performed for OS and DFS of included patients, respectively. For DFS, the univariate analysis indicated that the high TK1 increased the risk of poor prognosis (HR = 2.38, 95% CI: 1.64–4.23, *P*=0.000). The Kaplan–Meier curve indicated the high TK1 group was poorer than that in the low TK1 group (*P*=0.002, Figure 1). The age >50 (HR = 1.12, 95% CI: 1.03–2.76, *P*=0.026), lymph node number (compared with non-lymph node, 1–5 for HR = 1.24, 95% CI: 0.95-3.21, *P*=0.054; 6–10 for HR = 1.16, 95% CI: 1.09–4.01, *P*=0.017; >10 for HR = 1.34, 95% CI: 1.19–2.34, *P*=0.000), advanced stage (HR = 1.71, 95% CI: 1.16–2.69, *P*=0.018), PVI present (HR = 4.07, 95% CI: 1.37–9.01, *P*=0.006), ER positive (HR = 0.25, 95% CI: 0.21–0.81, *P*=0.042) and PR positive (HR = 0.38, 95% CI: 0.24–0.75, *P*=0.012) were also correlated with the prognosis in breast cancers patients with T2DM. Others factors were not associated with DFS, including BMI, smoking, tumor size, histological type, surgery and chemotherapy, endocrine therapy (*P*>0.05). For the OS, similar results were found that the high TK1 was related to poor OS (HR = 1.89, 95% CI: 1.34–3.67, *P*=0.000). The age, lymph node number (compared with non-lymph node, 1–5 for HR = 1.38, 95% CI: 0.87–2.98, *P*=0.078; 6–10 for HR = 1.28, 95% CI: 1.14–3.65, *P*=0.032; >10 for HR = 1.64, 95% CI: 1.27–4.56, *P*=0.000), advanced stage (HR = 1.76, 95% CI: 1.24–3.15, *P*=0.020), PVI present (HR = 3.78, 95% CI: 1.82.65–6.27, *P*=0.000), ER (HR = 0.34, 95% CI: 0.28–87, *P*=0.024) and PR positive (HR = 0.26, 95% CI: 0.17–0.74, *P*=0.042) were also associated with OS. The Figure 2 presented the Kaplan–Meier curve and the high TK1 showed a poorer OS (*P*=0.000).

**Table 2:** Univariable Cox regression analysis of DFS and OS for patients with breast cancer

Parameter	DFS		OS	
	HR (95% CI)	*P*	HR (95% CI)	*P*
Age (>50 vs ≤50)	1.12 (1.03–2.76)	0.026	1.23 (1.22–3.56)	0.021
BMI ≥ 24	1.43 (0.85–2.64)	0.234	1.57 (0.61–2.97)	0.317
Smoking (yes vs no)	1.37 (0.91–3.12)	0.381	1.48 (0.62–2.87)	0.406
Tumor size > 2 cm	1.92 (0.63–2.74)	0.419	2.02 (0.86–3.19)	0.602
Lymph node metastases	1.38 (1.19–2.64)	0.000	2.19 (1.81–3.92)	0.000
Stage				
II vs I	1.25 (0.73–2.54)	0.814	1.38 (0.61–2.85)	0.754
III vs I	1.71 (1.16–2.69)	0.018	1.76 (1.24–3.15)	0.020
Lymph node number (*n*, %)				
0	1.00		1.00	
1–5	1.24 (0.95–3.21)	0.054	1.38 (0.87–2.98)	0.078
6–10	1.16 (1.09–4.01)	0.017	1.28 (1.14–3.65)	0.032
>10	1.34 (1.19–2.34)	0.000	1.64 (1.27–4.56)	0.000
PVI present	4.07 (1.37–9.01)	0.006	3.78 (2.65–6.27)	0.000
ER positive	0.25 (0.21–0.81)	0.042	0.34 (0.28–0.87)	0.024
PR positive	0.38 (0.24–0.75)	0.012	0.26 (0.17–0.74)	0.042
HER2 positive	0.59 (0.28–3.26)	0.102	0.41 (0.26–1.97)	0.213
Ki-67	1.67 (0.92–2.86)	0.248	1.71 (0.49–3.12)	0.465
Endocrine therapy (no vs yes)	1.24 (0.64–3.12)	0.412	1.61 (0.79–4.10)	0.347
Histological type				
Others	1.00		1.00	
Non-infiltrated type	1.08 (0.54–2.16)	0.321	1.19 (0.64–4.12)	0.501
Infiltrated type	1.23 (0.65–6.12)	0.468	1.27 (0.31–3.48)	0.699
Conservative surgery	0.63 (0.24–3.18)	0.514	0.67 (0.34–3.41)	0.476
Chemotherapy	1.36 (0.57–3.16)	0.716	1.74 (0.69–4.01)	0.715
TK1 (high vs low)	2.38 (1.64–4.23)	0.000	1.89 (1.34–3.67)	0.000

**Table 3:** Multivariable Cox regression analysis of DFS and OS for patients with breast cancer

Parameter	DFS		OS	
	HR (95% CI)	*P*	HR (95% CI)	*P*
PVI present	2.17 (1.19–4.08)	0.020	2.22(1.89–3.27)	0.010
PR positive	0.26 (0.23–0.71)	0.030	-	-
Lymph node number (*n*, %)				
0	1.00		1.00	
1–5	1.17 (0.36–1.38)	0.067	1.26 (0.71–2.56)	0.102
6–10	1.26 (1.04–3.65)	0.033	1.18 (1.06–3.13)	0.024
>10	1.28 (1.12–2.09)	0.024	1.55 (1.34–3.87)	0.010
TK1 (high vs low)	1.83 (1.22–3.17)	0.001	1.63 (1.19–2.08)	0.006
Stage				
III vs II	1.51 (1.28–2.47)	0.032	1.46 (1.15–2.38)	0.029

Abbreviation: RDW, red cell distribution width.

The multiple Cox regression analysis was presented in Table 3. The multivariate Cox regression indicated that TK1 was still associated with DFS (HR = 1.83, 95% CI: 1.22–3.17, *P*=0.001) and OS (HR = 1.63, 95% CI: 1.19–2.08, *P*=0.006), including lymph node number, stage, PVI and PR positive (*P*<0.05).

## Discussion

Circulating nucleic acids are being recognized as potential predictive biomarkers of response to therapies due to their specificity and lack of invasiveness of sampling procedures [15]. We investigated whether exosome mRNA expression of TK1 could be used as biomarker of prognosis in the biomarker study. The present study indicated that the high serum TK1 was associated with poor OS and DFS in breast cancer patients with T2DM. The high serum TK1 was an independent predictor of poor survival in breast cancer patients with T2DM. Our findings indicated that the clinical monitoring should be performed for breast cancer.

At present, the serum tumor markers of breast cancer are mainly with carcinoembryonic antigen (carcinoma-embryonic antigen, CEA), carbohydrate antigen 15-3 (cancer antigen15-3, CA15-3), carbohydrate antigen-125 (cancer antigen-125, CA-125) and tissue polypeptide specific antigen (tissue polypeptide-specific antigen, TPS) etc. [16]. It has certain value in the diagnosis, treatment effect, prognosis and recurrence of breast cancer. However, due to the non-specific tumor markers of organs, their sensitivity and specificity are not good [17]. Therefore, it is necessary to search for tumor markers related to breast cancer with high sensitivity and good specificity to provide evaluation basis for the efficacy of radiotherapy for breast cancer. TK1 is a special enzyme that is involved in cell cycle regulation and affects cell division [18]. In healthy adults, TK1 levels are so low that they are barely detectable. However, with abnormal cell proliferation and cancellation, TK1 levels increased sharply. At present, studies have proved that TK1 expression level is positively correlated with tumor load in gastric cancer [19], lung cancer [20], early breast cancer and other cancers [21]. TK1 is a key enzyme for thymine nucleoside DNA synthesis and its activity is closely related to cell proliferation cycle. TK1 activity in the dormant phase (G_0_ phase) is very low and almost undetectable. When cells transition from G_1_ phase to S phase, TK1 gradually increases and reaches its peak in S phase. When cell division ends, TK1 gradually degrades in cells. Therefore, when normal cells divide, there is very little TK1 in the body, while malignant tumor cells proliferate vigorously, and the regulation factors of cell cycle are out of balance, losing the normal periodicity, TK1 increases significantly correspondingly. Therefore, TK1 can be used as a marker to reflect the growth and proliferation degree of tumor cells [22]. He et al. found that the serum TK1 expression level of breast cancer patients were higher than that of healthy adult females, indicating that TK1 plays an important role in the occurrence and development of breast cancer [23]. Therefore, detection of serum TK1 level can be used as one of the auxiliary diagnostic tests for early breast cancer. The results of the present study showed that the positive rate of TK1 expression in breast cancer patients with tumor diameter > 2.0 cm was higher than those with tumor diameter ≤ 2.0 cm, suggesting that TK1 expression was correlated with tumor size in breast cancer patients. This finding was consistent with our results that the TK1 was associated with tumor size > 2 cm. TK1 gradually increased in the early stage of cell division and degraded in the cell after cell division. Therefore, after normal cell division, the expression level of TK1 is low, while in malignant tumors, abnormal cell proliferation increases and the negative feedback mechanism is disordered, and a large number of TK1 is released into the blood, resulting in an obvious increase in serum TK1 level [23]. Therefore, dynamic monitoring of the activity of TK1 is of certain clinical value in evaluating the tumor progression of breast cancer patients. Our results indicated the TK1 was a biomarker of poor prognosis in breast cancer and provided some support for theory.

Studies on the relationship between TK1 expression in tissues and clinicopathological factors and other tumor markers of breast cancer have been reported, but the results still remain controversial. In 2001, TKI expression in cancer tissues of 1692 breast cancer patients was analyzed, and it was found that TKI level was related to tumor size, tissue grade and ER expression [24]. In 2004, He et al. analyzed the TKI expression in the cancer tissues of 54 patients with invasive ductal carcinoma, and found that the expression of TKI was positively correlated with the pathological grade and clinical stage of the tumor [25]. In 2010, Chen et al. [26] examined the expression of Ki-67 and TK1 in 89 cases of breast cancer tissues and found that TK1 expression was associated with lymph node metastasis, TNM stage and histological grade, had nothing to do with patients’ age, but they also found that TKI there was no significant correlation with Ki-67 expression. They assumed that TK1 and Ki-67 probably are two independent proliferation index of breast cancer cells [26]. The present study confirmed the relationship in serum expression levels.

The primary strength is that the present study consisted of a large sample size with adequate data analysis. There are still several study limitations. First, the study population was restricted in breast cancer patients with T2DM. The interpretation of results should be cautious when being applied in other population settings. Second, previous studies examined the TK1 expression in the cancer tissues and we detected the TK1 levels in the serum. This is different in different samples. Third, the present study explored the relationship between serum TK1 level and prognosis in clinical populations. Finally, the glycemic control is different for each patient, we cannot further make some estimations for these data, which may affect the results. We assumed there are no differences within patients. Beside, we did not explore the specific molecular mechanism. Further research is required.

In conclusion, our study found that high pretreatment serum TK1 levels in breast cancer patients was associated with poor OS and DFS. TK1 could be a potential predictive factor in differential diagnosis of poor prognosis from all patients. Future studies should explore the specific molecular mechanism and focused on long-term outcomes. Patients may benefit from regular clinical surveillance for TK1.

## Abbreviations

AC-T: doxorubicin or epidubicin - cyclophosphamide
BMI: body mass index
CI: confidence interval
DFS: disease-free survival
DNA: deoxyribonucleic acid
ER: estrogen receptor
FEC: fluorouracil medoxorubicin and cyclophosphamide
5-Fu: 5-Fluorouracil
HR: hazard risk
Ki-67: cell proliferation index Ki-67
KPS: Karnofsky performance status
OS: overall survival
PR: progesterone receptor
PVI: peritumoral vascular invasion
T2DM: type 2 diabetes mellitus
TEC: docetaxel rubicin cyclophosphamide
TK1: thymidine kinase 1
